# Analysis of Italian requests for compensation in cases of responsibility for healthcare-related infections: A retrospective study

**DOI:** 10.3389/fpubh.2022.1078719

**Published:** 2023-01-05

**Authors:** Maricla Marrone, Pierluigi Caricato, Federica Mele, Mirko Leonardelli, Stefano Duma, Ettore Gorini, Alessandra Stellacci, Davide Fiore Bavaro, Lucia Diella, Annalisa Saracino, Alessandro Dell'Erba, Silvio Tafuri

**Affiliations:** ^1^Section of Legal Medicine, Department of Interdisciplinary Medicine, Aldo Moro University of Bari, Bari, Italy; ^2^Attorney of Supreme Court, Department of Economics and Finance, Aldo Moro University of Bari, Bari, Italy; ^3^Department of Precision and Regenerative Medicine and Ionian Area, Clinic of Infectious Diseases, Aldo Moro University of Bari, Bari, Italy; ^4^Section of Public Health, Department of Interdisciplinary Medicine, Aldo Moro University of Bari, Bari, Italy

**Keywords:** healthcare-related infections, surgical site infection, health malpractice, risk management, health responsibility

## Abstract

**Introduction:**

The aim of this study was to examine the type of compensation claims for alleged medical malpractice in the field of healthcare-related infections in Italy.

**Methods:**

It was analyzed which was the most frequent clinical context, the characteristics of the disputes established, which were the alleged damages most often complained of, which were the possibly censurable behaviors of the health professionals, and which were the reasons for acceptance or rejection of the request for compensation.

**Results:**

In 90.2%, the issue questioned regarded surgical site infections. The most common pathogens involved were coagulase-negative Staphylococci (34.1%) and *Staphylococcus aureus* (24.4%). The lack or non-adherence to protocols of prophylaxis and/or prevention of healthcare-related infections was the most reported cause of acceptance of the request of compensation.

**Discussion:**

According to our data, a stronger effort should be made in terms of risk management perspective in order to ensure the develop and application of protocols for prevention of Gram-positive healthcare-related infections and strengthen infection control and antimicrobial stewardship programs.

## Introduction

Hospital or nosocomial infections, also called healthcare-related infections (HAIs) are defined as infections acquired during the hospitalization, not incubating during the hospital admission, and occurring at least 48 h after admission. Infections arising after discharge but resulting from hospitalization are also considered HAIs (1).

Surgical site infection (SSI) represents the most frequent type of HAIs in post-surgical hospitalizations and is burdened by high mortality rates, lengthened hospitalization times, need for intensive treatments, and need of hospital readmission (2). However, the incidence of SSIs has gradually decreased over time thanks to the prevention activities that have been implemented in healthcare facilities (3–5).

Hospital infection prevention and containment activities are part of the quality improvement actions and are essential to ensure patient safety (6). Healthcare facilities adopt infection prevention protocols by applying risk reduction interventions related to exposure to healthcare (7). The results of the Study of the Efficacy of Nosocomial Infection Control highlighted that the application of surveillance models can lead to a significant decrease in nosocomial or healthcare-related infections (8).

In recent years, the increase attention to the prevention of healthcare-related infection has allowed the creation of official protocols, supervised by doctors and/or trained health personnel. The management by accreditation teams, the increase of internal hospital protocols, the impact on hospital reimbursements, and the transparency of the dissemination of infection-related outcomes have allowed infection prevention protocols to become a cornerstone of healthcare in all areas.

Furthermore, the implementation models have increasingly focused on the prevention of infection rather than on its monitoring, with the progressive creation of working groups focused on the exclusive prevention of “healthcare-related” infection. At the same time, since healthcare has gradually decentralized from the hospital environment to the community, the concept of health epidemiology was born in order to define the multiple preventative actions that can be implemented in different healthcare realities.

Despite the diverse types of organizations found in the various Countries, the members of the infection prevention group have the task of implementing initiatives aimed at improving quality and ensuring patient safety, led by a team leader in direct contact with the hospital management (9).

In Italy, in order to ensure territorial uniformity in the management of nosocomial infections, the Circular of the Ministry of Health 52/1985 “Fight against hospital infections” was issued. It set up a technical commission responsible for the fight against HAIs with different tasks and purposes (define the strategy, verify the effective application of surveillance and control programs and their effectiveness, and take care of the cultural and technical training of the hospital staff).

According to this Circular, the committee must be made up of personnel specialized in microbiology, infectious diseases and hygiene, whose actions are managed by the Medical Director (10).

The subsequent Circular of the Ministry of Health 8/1988 underlines how passive surveillance, i.e., based on notification systems, does not represent an effective method for HAIs surveillance. Active surveillance must therefore be implemented: this could be proposed in different forms, depending on the type of healthcare facility.

Dell'Erba et al. (11) assess that the specialist in forensic medicine should be part of the technical commission for the fight against hospital infections. In fact, due to the growing judicial litigation in the field of health responsibility and hospital infections, it seems clear that forensic, due to specific training, is the most suitable figure to direct the complex clinical risk management process. In fact, no other figure has the responsibility of the complex ethical and deontological implications of the problem, including those related to informed consent and to the patient's information about the risk of acquiring a nosocomial infection (12).

In the following years, because of the importance that HAIs play in Public Health, the issue was the subject of specific documents such as the Compendium of measures for the control of HAIs and the Recommendations for the control the nosocomial spread of methicillin-resistant *Staphylococcus aureus* (MRSA). In addition, the National Prevention Plan 2014–2018 and the National Antimicrobial Resistance Contrast Plan 2017–2020 reports its importance (13).

On this basis, this study aims to examine the type of compensation claims for alleged medical malpractice in the field of hospital infections in Italy. Moreover, in order to confirm or deny the results, authors have expanded, the case history of a recent work concerning claims for compensation for hospital infections (10).

On the basis of the dataset, it was analyzed which was the most frequent clinical context, the characteristics of the disputes established, which were the alleged damages most often complained of, which were the possibly censurable behaviors of the health professionals, and, above all, which were the reasons for acceptance or rejection of the request for compensation. We have also interpreted these results according to the preventive logic inherent in the management of clinical risk.

## Materials and methods

We conducted a retrospective study using the Portal of Telematic Services of the Ministry of Justice. This is a search engine that allows the search of sentences.

We randomly selected 41 judgments issued from 2020 to 2021 in Italy and concerning claims for healthcare-related infections. Keywords for the sentences' selections were: “hospital infection,” “nosocomial infection” and “health responsibility.” The content of the sentences was therefore examined in detail.

The most important characteristics of each sentence were considered, in addition to the competent Court and the date of the sentence, the sex and age of the plaintiff/appellant, the type of hospital infection, the pathogenic microorganism responsible for the infection, the type of intervention/treatment suffered by the patient, the outcome of the dispute and the reason for the sentence. These data are shown in Table 1.

**Table 1:** Request for compensation 2020–2021 in Italy.

**Court**	**Gender-age range of the applicant**	**Types of infection**	**Further specifications**	**Type of intervention / treatment**	**Outcome judgment**	**Reason for judgment**
Court of Bologna - 2021	M – N.D.	Prosthesis infection	*Staphylococcus epidermidis*	Knee arthroplasty	Acceptance of the application	Lack or non-adherence to protocols of prophylaxis and/or prevention of nosocomial infection
Court of L'Aquila - 2020	M – N.D.	Post-surgical infection	*Klebsiella pneumoniae*	Placement of ventricular drainages	Rejection of the application	No recognition of the cause link
Court of Rome - 2020	F – 75–80	Post-surgical infection	*Staphylococcus* (not specificied)	Excision of neoplasm	Rejection of the application	No recognition of the cause link
Court of Vicenza - 2020	F – 50–55	Post-surgical infection	*Staphylococcus con methicillin-resistant*	Arthrodesis	Acceptance of the application	Delay in diagnosis
Court of Salerno - 2020	F – N.D.	Post-surgical infection	*Pseudomonas aeruginosa + tafilococcus aureus*	Leg osteosynthesis	Acceptance of the application	Lack or non-adherence to protocols of prophylaxis and/or prevention of nosocomial infection
Court of Salerno - 2021	F – N.D.	Prosthesis infection	*Staphylococcus epidermidis + Aspergillus flavus + Acinetobacter baumannii*	Knee arthroplasty	Acceptance of the application	Lack or non-adherence to protocols of prophylaxis and/or prevention of nosocomial infection
Court of Ravenna - 2020	M – 65–70	Prosthesis infection	*Staphylococcus hemoliticus*	Knee arthroplasty	Acceptance of the application	Lack or non-adherence to protocols of prophylaxis and/or prevention of nosocomial infection
Court of Locri - 2020	F – N.D.	Post-surgical infection	*Candida* (not specificied)	Heart valve replacement	Acceptance of the application	Delay in diagnosis
Court of Firenze – 2021	M – N.D	Post-surgical infection	*Staphylococcus aureus*	Knee arthroplasty	Acceptance of the application	Lack or non-adherence to protocols of prophylaxis and/or prevention of nosocomial infection
Court of l'aquila - 2021	M– N.D.	Post-surgical infection	*Staphylococcus aureus*	Leg osteosynthesis	Rejection of the application	No recognition of the cause link
Court of Savona - 2021	F – N.D	Post-surgical infection	*Staphylococcus epidermidis*	Breast implant replacement	Rejection of the application	No recognition of the cause link
Court of Rome - 2020	M– 60-65	Other nosocomial infection	*Staphylococcus* (not specificied) *klebsiella*	Post traumatic bed rest	Rejection of the application	No recognition of the cause link
Court of Milan - 2021	M - N.D	Post-surgical infection	N.D.	Crystalline substitution	Acceptance of the application.	Lack or non-adherence to protocols of prophylaxis and/or prevention of nosocomial infection
Court of Rome - 2020	M - N.D	Post-surgical infection	*Staphylococcus aureus*	Rectal polyp removal	Acceptance of the application	Lack or non-adherence to protocols of prophylaxis and/or prevention of nosocomial infection
Court of Rome - 2020	M – N.D.	Post-surgical infection	*Acinetobacter emoliticus; Escherichia coli; Haemolyticus*	Hemicolectomy	Rejection of the application	No recognition of the cause link
Court of Torino - 2021	F – N.D	Post-surgical infection	*Klebsiella* (not specificied); *Enterobacter aerogens*	Cerebral vascular malforation excision	Acceptance of the application	Lack or non-adherence to protocols of prophylaxis and/or prevention of nosocomial infection
Court of Genova – 2020	M- N.D	Post-surgical infection	*Klebsiella pneumoniae*	Ureteroscopy	Acceptance of the application.	Lack or non-adherence to protocols of prophylaxis and/or prevention of nosocomial infection
Court of Viterbo - 2021	M - 0	Other nosocomial infection	*Escherichia coli*	Acute meningitis	Acceptance of the application.	Lack or non-adherence to protocols of prophylaxis and/or prevention of nosocomial infection
Court of Perugia - 2021	M – 55–60	Post-surgical infection	*Staphylococcus aureus*	Meniscectomy	Acceptance of the application.	Lack or non-adherence to protocols of prophylaxis and/or prevention of nosocomial infection
Court of Perugia - 2020	F – N.D	Post-surgical infection	N.D.	Knee arthroplasty	Acceptance of the application.	Lack or non-adherence to protocols of prophylaxis and/or prevention of nosocomial infection
Court of Rome - 2021	M – 80–85	Post-surgical infection	*Acinetobacter; Klebsiella pneumoniae; Staphylococcus aureus*	Excision of brain tumor	Acceptance of the application.	Lack or non-adherence to protocols of prophylaxis and/or prevention of nosocomial infection
Court of Ravenna - 2021	M – N.D	Post-surgical infection	*Staphylococcus epidermidis; Escherichia coli; Klebsiella pneumoniae*	Heart valve replacement	Rejection of the application	No recognition of the cause link
Court of Firenze - 2021	M – N.D	Post-surgical infection	*Staphylococcus saprofiticus*	Knee arthroplasty	Acceptance of the application.	Lack or non-adherence to protocols of prophylaxis and/or prevention of nosocomial infection
Court of Rome - 2020	F – 75-80	Post-surgical infection	N.D.	Vertebral decompression	Rejection of the application	No recognition of the cause link
Court Of Perugia - 2020	F – 55–60	Post-Surgical Infection	*Staphylococcus aureus Mrsa*	Treatment Of Hallux Valgus	Acceptance Of The Application.	Lack or non-adherence to protocols of prophylaxis and/or prevention of nosocomial infection
Court of Catania - 2020	F – 15–20	Prosthesis infection	*Staphylococcus* (not specificied)	Breast lift	Acceptance of the application.	Lack or non-adherence to protocols of prophylaxis and/or prevention of nosocomial infection – inadequate therapy
Court of Palermo - 2020	F – 70–75	Prosthesis infection	N.D.	Knee arthroplasty	Acceptance of the application.	Lack or non-adherence to protocols of prophylaxis and/or prevention of nosocomial infection
Court of Latina - 2020	F - N.D.	Post-surgical infection	*Staphylococcus epidermidis*	Knee arthroplasty	Acceptance of the application.	Lack or non-adherence to protocols of prophylaxis and/or prevention of nosocomial infection
Court of Milano - 2020	F - N.D.	N.D.	*Pseudomonas aeruginosa*	N.D.	Acceptance of the application.	Lack or non-adherence to protocols of prophylaxis and/or prevention of nosocomial infection
Court of Brindisi - 2020	M - N.D.	Other nosocomial infection	*Klebsiella pneumoniaea*	Intestinal excision	Rejection of the application	No recognition of the cause link
Court of Milano - 2020	M – 35–40	Prosthesis infection	*Escherichia coli*	Gluteoplastic	Acceptance of the application.	Lack or non-adherence to protocols of prophylaxis and/or prevention of nosocomial infection
Court of Pistoia - 2020	F – 55–60	Post-surgical infection	*Pseudomonas aeruginosa*	Cerebral malformation excision	Acceptance of the application.	Lack or non-adherence to protocols of prophylaxis and/or prevention of nosocomial infection
Court of Catania - 2020	F – 35–40	Post-surgical infection	N.D.	Foreign body removal	Acceptance of the application.	Lack or non-adherence to protocols of prophylaxis and/or prevention of nosocomial infection – inadequate therapy
Court of Torino - 2020	F – 80–85	Post-surgical infection	*Citrobacter freundii*	Crystalline substitution	Acceptance of the application.	Lack or non-adherence to protocols of prophylaxis and/or prevention of nosocomial infection
Court of Vicenza – 2021	F – 55–60	Post-surgical infection	*Serratia marcescens*	Brain tumor excision	Acceptance of the application.	Lack or non-adherence to protocols of prophylaxis and/or prevention of nosocomial infection
Court of Lecce - 2021	M - N.D.	Post-surgical infection	*Acinetobacter baumanii*	Cholecystectomy	Acceptance of the application.	Lack or non-adherence to protocols of prophylaxis and/or prevention of nosocomial infection
Court of Milano - 2021	F - N.D.	Prosthesis infection	*Staphylococcus aureus*	Knee arthroplasty	Acceptance of the application.	Lack or non-adherence to protocols of prophylaxis and/or prevention of nosocomial infection
Court of Palermo - 2021	F – 60–65	Post-surgical infection	*Staphylococcus aureus*	Arm osteosynthesis	Acceptance of the application.	Inadequate therapy
Court of Rieti - 2021	F – 60–65	Prosthesis infection	*Staphylococcus epidermidis*	Femoral osteosynthesis	Rejection of the application	No recognition of the cause link
Court of Firenze - 2021	M - N.D.	Prosthesis infection	*Staphylococcus aureus*	Pacemaker implant	Acceptance of the application.	Delay in diagnosis
Court of Bologna - 2021	F - N.D.	Post-surgical infection	*Staphylococcus* (not specificied)	Knee arthroplasty	Acceptance of the application.	Lack or non-adherence to protocols of prophylaxis and/or prevention of nosocomial infection

ND, not determined.

The content of judgement and judge's decisions.

Due to the severity of the Italian legislation on privacy and protection of personal data, it was not possible, in some cases, to infer some data, such as the registry data (e.g., age), of little relevance for the purpose of our discussion. However, in all the sentences, the motivation of the Judge's decision was exhaustively reported.

### Data analysis

Statistical analysis was conducted using Microsoft Excel 2013 software (Microsoft Corporation Redmond, WA, USA) and IBM SPSS Statistic version 25 for Windows (IBM Corporation, Armonk, NY, USA). The categories examined were represented in percentage terms.

## Results

We examined forty-one judgments issued by the relevant jurisprudence in Italy from 2020 to 2021 and relating to cases alleged health malpractice in the context of infections contracted in a hospital setting. Of all cases, 19 were brought by males (46.3%), while 22 by females (53.7%).

**Figure 1:**
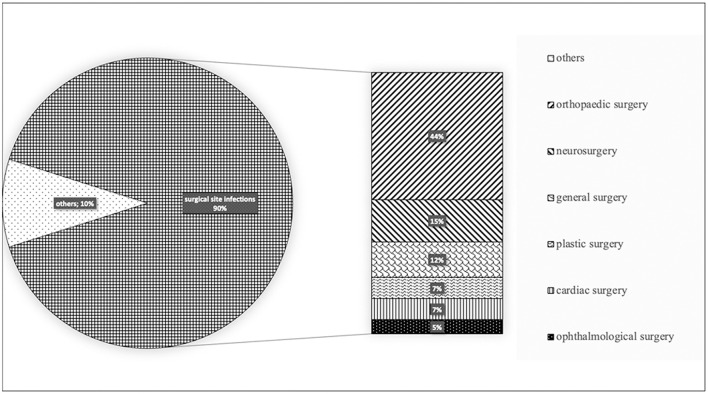
Types of nosocomial infections divided by hospital wards.

For 37 cases out of 41 (90.2%) the judgement regarded surgical site infections. By assessing surgical interventions, 18 (41%) were orthopedic, 6 (15%) neurosurgical, 5 (10%) general surgery, 3 (7%) plastic surgery, 3 (7%) cardiac surgery, 2 (5%) ophthalmological (Figure 1).

**Figure 2:**
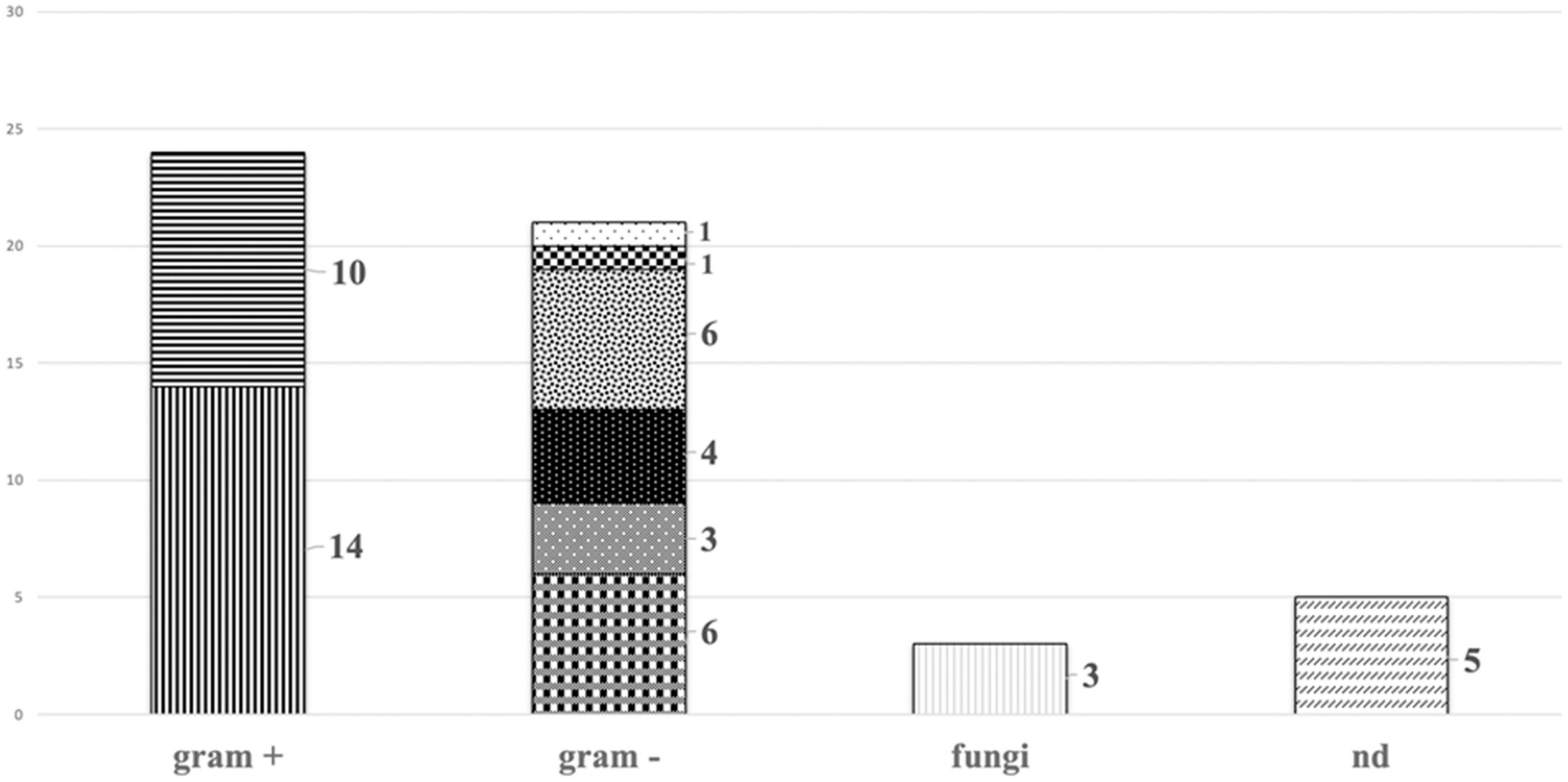
Types of pathogens.

The most common pathogens involved were Gram positive bacteria belonging to *Staphylococcus* spp., 14 coagulase-negative Staphylococci (34.1%) and 10 *Staphylococcus aureus* (24.4%). The remaining were Gram negative bacteria, including multidrug resistant pathogens−6 (14.6%) *Klebsiella pneumoniae*, 3 (7.3%) *Pseudomonas aeruginosa* and 4 (9.8%) *Acinetobacter baumannii*-; 6 (14.6%) infections were caused by *Escherichia coli*, 1 (2.4%) *Citrobacter freundii*, 1 (2.4%) *Serratia marcescens*. In addition, 3 (7.3%) were caused by *Candida* spp. The pathogen was not isolated in 5 cases (12.2%). Polymicrobial infections were diagnosed in 6 cases (14.6%) (Table 2, Figure 2).

**Table 2:** Request for compensation 2020–2021 in Italy.

**Pathogens**	***N***	**%**
Coagulase – negative Staphylococci	14	34.1
*Staphylococcus aureus*	10	24.4
*Klebsiella pneumoniae*	6	14.6
*Pseudomonas aeruginosa*	3	7.3
*Acinetobacter baumanii*	4	9.8
*Escherichia coli*	6	14.6
*Citobacter freundii*	1	2.4
*Serratia marcescens*	1	2.4
*Candida* spp	3	7.3
Polymicrobial infections	6	14.6
N.d.	5	12.2

Nd, not determined.

Types of pathogens.

**Figure 3:**
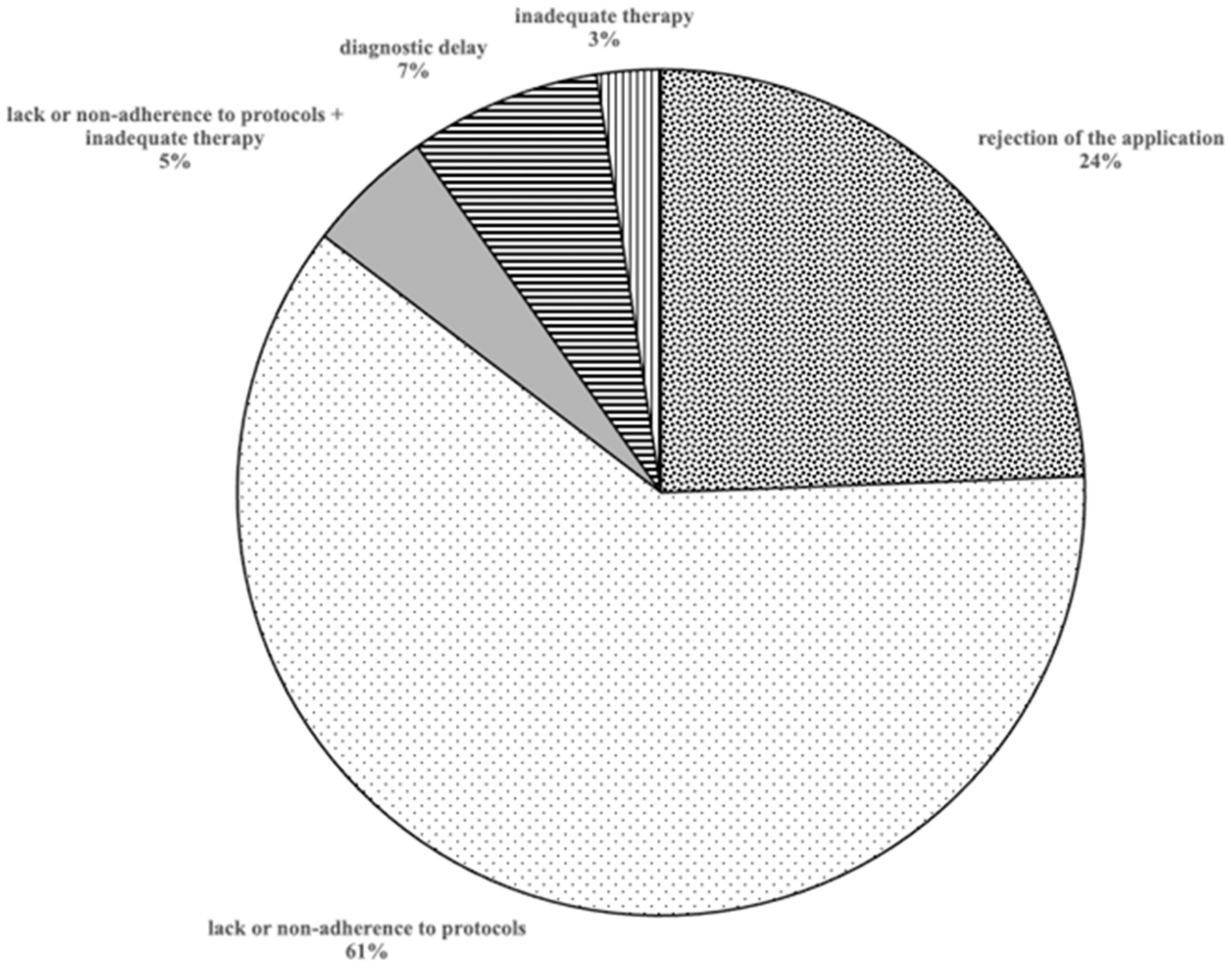
Reasons of acceptance or rejection of the requests of compensations.

The judgments examined resulted in the claim of the plaintiff being accepted in 31 cases (75.6%) and in the rejection of the claim in 10 cases (24.4%) (Figure 3).

Analyzing the group of 24 infections caused by Gram-positive pathogens (3 polymicrobial), 17 (71%) were accepted: in 14 cases (82.4%) the reason for acceptance was the lack or non-adherence to protocols of prophylaxis and/or prevention of nosocomial infection; in 2 cases (11.8%) and 1 case (5.9%) due to delay in diagnosis or inadequate therapy, respectively.

Among the 13 cases of infections caused by multidrug resistant organism, 9 (69.2%) were accepted due to the lack or non-adherence to protocols of prevention of nosocomial infection.

Finally, among the remaining, 9 were accepted: 8 (88.9%) due to lack or non-compliance to protocols and 1 (11.1%) due to diagnostic delay (Table 3).

**Table 3:** Request for compensation 2020–2021 in Italy.

	**Gram positive pathogens (%)**	**Multidrug resistant pathogens (%)**	**Others pathogens (%)**
Lack or non-compliance to protocols	14 (82.4%)	9 (69.2%)	8 (88.9%)
Diagnostic delay	2 (11.8%)	/	1 (11.1%)
Inadequate therapy	1 (5.9%)	/	/

Acceptance of the requests divided by pathogens.

## Discussion

This works revised a series of sentences by various Italian Courts regarding surgical site/hospital acquired infection, in order to identify the major causes of acceptance of claims. In general, the lack or non-adherence to protocols of prophylaxis and/or prevention of nosocomial infection was the predominant cause of acceptance, while diagnostic delay or inadequate therapy caused a residual part of acceptance.

This case series is in line with previous studies showing that the majority of surgical site infections are caused by *Staphylococcus* spp, in particular by Staphylococcus aureus (14), both methicillin-susceptible (MSSA) and methicillin-resistant *Staphylococcus aureus* (MRSA), that are burdened by high rate of mortality and healthcare costs (15).

Consequently, bundles for prevention of *Staphylococcus aureus*-SSI (SA-SSI) for patients undergoing major clean surgery, including cardiothoracic and orthopedic devices implantation have been studied and implemented in several facilities (16, 17).

These preventive strategies are based on the following points: identification of SA carriers by nasal swab and de-colonization of carriers with mupirocin (intranasal) and chlorhexidine (for skin and hair) (18, 19); pre-operative prophylaxis including vancomycin for patients colonized by MRSA (20); education of healthcare personnel on hand hygiene and contact isolation for colonized patients.

Hence, given the strengthened of evidence about the efficacy of preventive measure for reduction of risk for SA-SSI, the neglect in adoption of protocol exposes the clinicians to sentences of responsibility for nosocomial infection. However, if a SA-SSI occurs, despite the proper implementation of strategies for infection prevention and control, health practitioners are exempted from responsibility; indeed, it should be considered that up to 55–70% of hospital acquired infection are potentially preventable (21).

Despite Gram positive bacteria are the mainly involved pathogens, even Gram-negative bacteria including *Enterobacterales* and non-fermentative rods may cause surgical site infections in cardiac surgery (22) and orthopedic surgery (23).

In particular, the diffusion of multidrug resistant Gram-negative bacteria (MDR-GNB) pose a high risk of SSI, increasing mortality rate, length of hospital stays, legal disputes and, in turn, costs. Indeed, standard antibiotic prophylaxis may be ineffective against Gram negative pathogens, especially in case of MDR-GNB. Still, protocols for prevention of GNB SSI are lacking, especially in Italy.

For instance, in the field of colorectal surgery, a carbapenem-based prophylaxis for carriers of extended spectrum Beta-Lactamase producing Enterobacteriaceae, usually unresponsive to common prophylaxis, showed to be effective in reducing incidence of SSI, if compared with standard protocols (24). However, the benefit of this approach in other setting is unknown, and standard prophylaxis with cephalosporins in non-colorectal surgery in patients with rectal colonization by ESBL could be considered appropriate. Beyond the choice of an appropriate antibiotic, the route of administration of antibiotic drug plays an important role in the effectiveness of prophylaxis. According to current literature, the antibiotic should be administered intravenously within 60–120' before skin incision, in order to achieve adequate blood and tissue concentration during surgical procedure, considering half-life of molecules (25). In this regard, antibiotic redosing during surgical intervention lasting more than 2 times the half-life of drug is effective in lowering risk of SSI (26). In addition, the combination of oral and intravenous antibiotic prophylaxis has shown to be a viable approach in reducing rate of SSI in colorectal elective surgery (27). Still, antibiotic prophylaxis should be discontinued within 24–48 h after surgical procedure, because longer duration is not associated with a lower rate of SSI, but rather with increase of adverse events and increased risk of nosocomial infections (28). Nevertheless, despite the scarcity of protocols for prevention of GNB-SSI in major clean surgery, the clinicians are not free from responsibility in case of MDR infection, if measurements of stewardship and infection control are not properly applied.

Furthermore, if on the one hand the protocols for the prevention of nosocomial infections need to be implemented and continuously updated, also in relation to the biological variability of the microorganisms involved, on the other hand Health Facilities were often condemned as they were not able to provide documentary evidence of the use of these protocols. This occurred regardless of whether the doctors had actually taken steps to prevent the onset of hospital infection. In Italy the legal rules for sharing the burden of proof are stringent. The legal principles governing the responsibility of healthcare workers and the health facilities are linked to the “hospitalization contract.” In the case of non-compliance, the provisions of art. 1,218 “liability of the debtor” and art. 1,228 “liability for acts auxiliaries” of the Civil Code were applied. On the basis of these dictates, Healthcare Facility must demonstrate that it has fulfilled all the specific obligations (disinfection, sterilization, hand washing, environmental monitoring) or, alternatively, the absence of the causal link between the alleged breach and damage (i.e., between the conduct and the occurrence of the infection). This principle constitutes an application of the art. 1,218 of the Italian Civil Code which divides the burden of proof in the contractual context to which governs civil litigation in the field of health liability.

However, due to the complexity of all the procedures, it is challenging for the structure to demonstrate that it has actually carried out the proper conduct.

Although infections might not be attributable to the hospital, once it is demonstrated that a patient has contracted a nosocomial infection, the hospital must prove the adoption of all the necessary measures for the correct sanitation, to avoid contamination of patients by nosocomial pathogens.

This aspect justifies the divergence between our cases and the results of civil trials for medical liability in Italy. Data extracted from Consulcesi, an Italian company operating in the field of legal health assistance and health professionals, report that about 66% of civil trials in the field of health responsibility in Italy are rejected (29). In our case series, however, only 24.4% of the cases examined resulted in a non-acceptance of the plaintiff's request.

According to ECDC surveillance atlas of infectious diseases of 2017, SSI occur after 2.4% of surgical intervention in Italy, while European incidence is 2.7 % (30). The rate of SSI may be further reduced throughout the implementation of national surveillance programs, with the aim to identify the appropriate intervention to reach the objective (31).

According to these considerations and our data, a stronger effort should be made in terms of risk management perspective in order to: ensure the application of documented protocols for prevention of SSI by Gram positive; develop protocols to prevent GNB-SSI, based on local and regional epidemiology and risk assessment; strengthen infection control and antimicrobial stewardship programs. The application of SSI prevention bundle, including pre-operative procedures (skin decolonization with mupirocin and chlorhexidine for SA carriers, appropriate administration of antibiotic prophylaxis, proper washing of healthcare personnel and surgical room), intraoperative procedures (adequate blood oxygenation, glycaemia, normothermia) is associated with lower risk of SSI. The implementation of educational programs for healthcare personnel, along with periodic performance monitoring and multidisciplinary audit could represent some applicable strategy to promote the application of good clinical practice for prevention of SSI (32, 33).

In this scenario, the involvement of patients in informative programs is of great relevance. Healthcare professionals should educate patients about infectious risk and foster correct behavior for its decrease, including preoperative shower, *S. aureus* decolonization, stop smoking, and correct wound care after surgery (34).

Additionally, other important strategies should be implemented at hospital-level to reduce the risk of hospital acquired infections: (i) infection control and preventions protocols in all wards (35); (ii) controlled prescription of antimicrobial therapies, especially those with high selective pressure (36); (iii) dedicated microbiological alert in case of severe infections, including bloodstream infections and/or detection of multidrug resistant pathogens (37).

Interestingly, by comparing data of incidence of surgical site infections (Table 4), based on European Center for Disease Prevention and Control (eCDC) surveillance data of the year 2017 (38), the incidence of infective complications after surgery may significantly differ between different Countries and different types of surgical procedures. This support the need of tailoring specific preventing strategies, in addition to aforementioned ones, according to local epidemiology.

**Table 4:** Incidence of surgical site infections per major types of surgical procedures in Italy and European countries.

**Type of surgical procedure**	**Incidence in Italy**	**State with lowest incidence in EU/EAA**	**State with higher incidence in EU/EAA**	**Incidence in EU/EAA**	**Difference (Italy vs. EU/EAA)**
**In-hospital incidence per 1,000 post-operative days (N/1,000)**					
Coronary artery bypass graft	1.3	Norway = 0.7	Lithuania = 3.2	1.2	0.1
Cholecystectomy	0.9	Lithuania = 0.5	England = 7.6	1.4	−0.5
Colon surgery	3.1	Lithuania = 2.7	Portugal = 10.2	4.9	−1.8
Cesarean section	0.1	Italy = 0.1	Estonia = 1.7	0.6	−0.5
Hip prosthesis	0.3	Finland = 0.2	Hungary = 0.9	0.3	0.0
Knee prosthesis	0.2	France = 0.1	Portugal = 0.4	0.1	0.1
Laminectomy	0.3	Germany = 0.2	Hungary = 2.2	0.4	−0.1
**Incidence of SSI per 100 operations**					
Coronary artery bypass graft	2.4	England = 2.2	Lithuania = 5.5	2.7	−0.3
Cholecystectomy	1.0	Lithuania = 0.4	England = 4.0	1.5	−0.5
Colon surgery	5.0	Lithuania = 3.9	Portugal = 16.2	8.5	−3.5
Cesarean section	0.5	Italy = 0.5	England = 5.3	1.9	−1.4
Hip prosthesis	0.8	Ireland = 0.4	Norway = 2.2	1.0	−0.2
Knee prosthesis	0.6	England = 0.2	Hungary = 2.7	0.5	0.1
Laminectomy	1.0	Ireland = 0.2	Hungary = 2.7	0.7	0.3

EU/EAA = European Union/ European Economic Area.

If this proactive approach is fundamental in public health, it would be equally useful to raise the awareness of healthcare personnel toward medico-legal issues. Furthermore, the Italian Law requires to demonstrate what has been done by the hospital and this consequently means that hospital must keep the complete documentation of the preventive activities.

Finally, to encourage a correct reactive approach to the nosocomial infection, the contribution of an infectious disease specialist in order to avoid that any infection could be simplistically charged to a health responsibility.

## Data availability statement

The original contributions presented in the study are included in the article/supplementary material, further inquiries can be directed to the corresponding authors.

## Author contributions

ASa, AD, ST, and MM contributed to conception and design of the study. PC and ML organized the database. SD performed the statistical analysis. FM, EG, ASt, DB, and LD wrote sections of the manuscript. All authors contributed to manuscript revision, read, and approved the submitted version.
